# A Perfected Food

**Published:** 1904-12

**Authors:** 


### A PERFECTED FOOD.

In treating anemia is it not true that our first thought, and that to which our instinct should naturally lead us, is a normal blood standard? That there is a deficiency of iron in the blood in most forms of anemia, is, of course, indisputable; and to endeavor to supply this lack by the administration of iron seems but a common-sense procedure. This practice would be sufficient if anemia were, in reality, nothing more than a condition of iron deficiency; but the profession realize now that the underlying costive factor is a disturbance of the process of nutrition and cell-proliferation, and that iron poverty is but one manifestation of this disorder. Ample proof of this fact has been presented to every doctor when he has observed how anemic conditions persist in spite of the long contined administration of the various preparations of iron. Here, then, iron preparations must be supplemented by such remedies or by such a remedy as has the ability to awaken the depressed nutritive and cell-proliferating process. To stimulate, tone up and supply perfect nutrition in all anemic conditions, I have found Bovinine to meet every indication par excellence.—JOHN GRIGGS, M.D.

